# Immunocapture of virions with virus-specific antibodies prior to high-throughput sequencing effectively enriches for virus-specific sequences

**DOI:** 10.1371/journal.pone.0216713

**Published:** 2019-05-09

**Authors:** Dennis Knierim, Wulf Menzel, Stephan Winter

**Affiliations:** Leibniz-Institute DSMZ—German Collection of Microorganisms and Cell Cultures, Plant Virus Department, Braunschweig, Germany; Oklahoma State University, UNITED STATES

## Abstract

Virus discovery based on high-throughput sequencing relies on enrichment for virus sequences prior to library preparation to achieve a sufficient number of viral reads. In general, preparations of double-stranded RNA or total RNA preparations treated to remove rRNA are used for sequence enrichment. We used virus-specific antibodies to immunocapture virions from plant sap to conduct cDNA synthesis, followed by library preparation and HTS. For the four potato viruses PLRV, PVY, PVA and PYV, template preparation by virion immunocapture provided a simpler and less expensive method than the enrichment of total RNA by ribosomal depletion. Specific enrichment of viral sequences without an intermediate amplification step was achieved, and this high coverage of sequences across the viral genomes was important to identify rare sequence variations. Using this approach, the first complete genome sequence of a potato yellowing virus isolate (PYV, DSMZ PV-0706) was determined in this study. PYV can be confidently assigned as a distinct species in the genus *Ilarvirus*.

## Introduction

High-throughput sequencing (HTS) has become a central element of virus discovery research, as it provides global insight into the virus composition of a (plant) sample [1, 2]. The genome sizes of plant viruses are relatively small; nevertheless, to achieve reliable genome coverage, the number of reads must be high to trace sequences that can be imperceptible within the enormous overrepresentation of host sequences [1, 3]. Preparation for HTS requires enrichment for viral sequences prior to sequencing to balance the ratio between viral and host sequences [1]. Here, we applied different strategies for enriching for viral sequences and evaluated each option for its feasibility, enrichment efficiency and universality, the amount of starting material required and cost.

One possibility for obtaining a large number of viral reads is to start with the specific extraction of small RNAs. Sequencing of virus-derived small RNA (smRNA) is widely used to detect viruses with both RNA and DNA genomes [4, 5, 3, 1, 2]. Antiviral defense in plants involves the degradation of viral RNA by DICER to 21–24 nt-long RNA populations. Sequencing of these smRNAs would allow the assembly of entire viral genome(s); although, in reality, because of the nonrandom distribution of these smRNAs over the genome, contigs often cover a portion of the viral genome at best, and the sequence reconstruction of complete genomes is rarely accomplished [6, 3, 7, 8]. Furthermore, repetitive sequences or sequence variations make it difficult to assemble complete genomes with a high level of confidence. However, the mapping of sequences to a reference genome leads to consistent genome coverage [2], whereas sequence gaps must be closed using conventional methods [5]. Due to these shortcomings, it has not been possible to establish this approach as a standard for HTS.

Theoretically, the genomes of RNA and DNA viruses can be analyzed with the genomic and/or mRNA sequences found in total RNA preparations [9, 10, 11, 12, 13, 14, 1]. Because viral RNA represents only a tiny fraction of total RNA, enrichment for virus sequences prior to de novo assembly is a precondition to facilitate cost-effective HTS of subsequent virus bioinformatics. By selectively depleting ribosomal RNA molecules using capture probes specific for abundant rRNAs, a large proportion of ribosomal RNA can be removed prior to cDNA synthesis, although some host RNA remains, which can considerably disturb subsequent viral analysis. When RNA preparations from virus-infected plants are subjected to HTS, it is possible to adjust the read length in the commonly used Illumina sequencing systems over a range of approximately 50 nt (Illumina HiSeq) to 300 nt (Illumina MiSeq) according to the chosen platform. Highly sensitive and efficient cDNA library preparation using, e.g., Nextera XT Preparation Kits (Illumina), combined with a long reading length (301 nt paired end reads) in HTS then allows the high-precision assembly of nearly full- to full-length virus genomes from minute amounts of template RNA [9, 10, 11].

The replication of plant RNA viruses involves dsRNA intermediaries, which are molecules that are otherwise very rare in plants. Because dsRNA is very stable and can be easily purified, the obtained dsRNA consists almost entirely of virus RNA in the sense and complementary sense direction and can serve as an ideal template for cDNA synthesis and HTS [15, 16, 1]. The drawback is that not all RNA viruses accumulate high concentrations of dsRNA. Furthermore, due to the different replication mechanisms of DNA viruses compared to RNA viruses, no full-length genomic dsRNA is produced as an intermediate replication product in DNA virus-infected plants [1]. The sequences of DNA viruses identified in dsRNA preparations most likely come from contaminating DNA or ssRNA that is still present in dsRNA preparations [17, 18, 19]. Although there are published dsRNA extraction protocols that require less starting material [20], standard dsRNA extraction protocols require several grams of plant tissue [21]. Similarly, while the extraction of RNA/DNA from purified virions may provide a high ratio of viral to host reads [7, 1], virus purification is cumbersome, requires a large number of virus-infected plants and is therefore not conducive to high-throughput methods. The use of VANA (virus-associated nucleic acids) may provide a better solution for the enrichment of virus sequences [22, 23].

Other enrichment methods for virus sequences correspond to particular virus genome characteristics. Plant viruses with polyadenylated RNA genomes can be enriched by the purification of plant mRNA [24, 25, 1]. Viruses with circular DNA genomes can be amplified in a rolling circle amplification (RCA) process that allows high-resolution analysis of the entire assemblage of DNA molecules associated with plant DNA viruses [12, 26].

Here, we present a method for capturing virions with virus-specific antibodies. With this method, random cDNA synthesis is directly performed using RNA from immunocaptured (IC) virions. We assessed the IC template preparation method for the HTS analysis of *potato leafroll virus* (PLRV, genus *Polerovirus*, family *Luteoviridae*), *potato virus S* (PVS, genus *Carlavirus*, family *Betaflexiviridae*) and *potato virus Y* (PVY, genus *Potyvirus*, family *Potyviridae*). To demonstrate the suitability of the IC-HTS method, we determined the complete genome sequence of potato yellowing virus (PYV), an unassigned species in family *Bromoviridae* for which no genome sequence was available.

## Materials and methods

### Virus isolates

The three viruses PLRV, PVS and PVY were present in a potato sample (W13-136) originating from a potato field in Lower Saxony in Germany (potato, *Solanum tuberosum* L. var. ‘Bamberger Hörnchen’, collected in 2013) and were kindly provided by Dr. Volker Zahn (Chamber of Agriculture of Lower Saxony, Germany). The PLRV isolate (PLRV-136) was separated from PVS and PVY by sequential aphid transmission via the intermediate host *D*. *stramonium* and back transfer to potato plants (‘Bamberger Hörnchen’). The potato yellowing virus isolate DSMZ PV-0706 was maintained at the Plant Virus Department in *D*. *stramonium*. This virus isolate was originally obtained from a pepino (*Solanum muricatum*) fruit purchased in a supermarket in Braunschweig (Lower Saxony, Germany) in 2001 and was kindly provided by Dr. D.E. Lesemann (JKI, Braunschweig). The geographic origin of the fruit was not specified.

### Antisera and ELISA tests

Antisera against PLRV (DSMZ AS-0741), PVA (DSMZ AS-0535), PVM (DSMZ AS-0273), PVS (DSMZ AS-0547), PVY (DSMZ AS-0343), PVX (DSMZ AS-0126) and PYV (DSMZ AS-0599) and their respective positive controls were taken from the stock of the DSMZ Plant Virus Department. All ELISA tests were performed following DSMZ standard procedures (www.dsmz.de).

### Illumina library preparation from total RNA

Illumina libraries were prepared from total RNA extracts as previously reported [11]. Total RNA extraction (RNeasy Plant Mini Kit, QIAGEN, Germany), removal of ribosomal RNA (RiboMinus Plant Kit, Invitrogen), random cDNA synthesis with random octamer primers (RevertAid H Minus Reverse Transcriptase, Thermo Fisher Scientific), second strand synthesis (NEBNext, mRNA Second Strand Synthesis Module, NEB), library preparation (Nextera XT Library Kit, Illumina, USA), DNA quantification (Qubit dsDNA HS Assay Kit, Life Technologies) and quality analyses (High Sensitivity DNA Chips, Agilent 2100 Bioanalyzer, Agilent Technologies) were performed using commercially available kits, essentially following the manufacturers’ protocols. All libraries were pooled and run as paired-end reads on a MiSeq sequencer (Illumina, 2x301) with the exception of Library-04 and Library-08, which were run on a NextSeq sequencer (Illumina, 2x151) (DSMZ, Germany).

### IC of virus particles for Illumina library preparation

IC was performed with magnetic sheep anti-rabbit IgG Dynabeads M-280 (Invitrogen) to capture virus-specific polyclonal antisera. Plant extracts were prepared by grinding fresh leaves (at 1:20 w/v) and freeze-dried leaf material (at 1:50 w/v) in standard ELISA sample extraction buffer (1x PBS, 0.5% Tween 20, 2% PVP-15 polyvinyl pyrrolidone, pH 7.4). For each IC reaction, a 1.5 ml Eppendorf tube was used, and 2 variants for coupling the magnetic beads with the target IgG and antigen (IC-1 and IC-2) were tested. For variant IC-1, 50 μl of purified beads was mixed with 1 ml of virus-infected plant extract and 4 μg of IgG. This mixture was incubated by shaking at 4°C overnight. For variant IC-2, 50 μl of purified beads was mixed with 4 μg of IgG diluted in 1 ml of sample extraction buffer without a virus-infected sample and incubated for 70 min at 37°C. Subsequently, the Dynabeads precoated with the IgGs were washed, and 1 ml of virus-containing plant extract was added, followed by overnight shaking at 4°C. For Library-03, all three antisera were mixed at a ratio of 1:1:1 (v/v/v) for a total of 4 μg/μl IgG.

Following the overnight incubation described above, the beads of IC-1 and IC-2 were washed with Dynabead washing buffer according to the manufacturer’s instructions. The bound virions were eluted from the beads with 69.5 μl of water and transferred to PCR tubes. Subsequently, 3 μl of random octamer primers (100 pmol/μl) and 0.5 μl of RiboLock RNase Inhibitor (Thermo Fisher Scientific) were added. After a denaturation step at 99°C for 2 min and immediate cooling on ice, 27 μl of master mix [2.5 μl of RevertAid H Minus Reverse Transcriptase, 20 μl of 5X Reaction Buffer, 0.5 μl of RiboLock RNase Inhibitor (all Thermo Fisher Scientific) and 4 μl of dNTP mix (25 mM each)] was added for cDNA synthesis, and the reaction was incubated at 45°C for 1 h, followed by inactivation at 70°C for 10 min. The synthesized cDNA was purified using a NucleoSpin Gel and PCR Clean-up kit (Macherey-Nagel). For library preparation, all subsequent steps were performed as described for the total RNA templates. As described above, the libraries were pooled and analyzed as paired-end reads.

### Sequence analysis

Sequence reads retrieved from the MiSeq platform were screened for plant virus sequences using Geneious 11.1.4 software. In the first step, all trimmed reads (error probability limit 0.01) from a library were mapped to the reference plant virus and viroid database (NCBI download 11.06.18) with a sensitivity of a maximum of 20% mismatches per read. Plant-related reads were removed before de novo assembly to reduce the number of nonspecific reads. Chloroplast (NC_008096) and chromosome (*S*. *tuberosum* v4.03, download from Phytozome 12) reads were removed from the potato samples; for the datura samples, only the chloroplast (NC_018117)-related reads were subtracted. De novo assembly was performed with the Geneious assembler (medium-low sensitivity/fast). The first 1000 contigs (starting with the greatest number of reads related to the respective contig) were compared by local Blastn searches against plant virus and viroid reference sequences, followed by Blastp searches against the plant virus protein reference database (NCBI download 11.06.18). Sequence analysis and alignments were carried out using the BLAST webserver (http://blast.ncbi.nlm.nih.gov/blast.cgi) and Clustal Omega (https://www.ebi.ac.uk/Tools/msa/clustalo/). Phylogenetic trees for PYV were inferred with 1,000 replicates of the neighbor-joining procedure, applying default settings using MEGA 6 [27].

To complete the viral genomes, 5’ and 3’ RACE experiments to verify the termini were performed for PLRV and the three genome components of PYV [28]. PCR fragments were amplified with Phusion Flash High-Fidelity PCR Master Mix (Thermo Fisher Scientific) and directly sequenced (HZI, Germany).

## Results

### Virus isolates

The identity of the virus isolates used and their purity were confirmed in principle by these investigations. The potato sample W13-136 tested positive for PLRV, PVS and PVY and negative for PVA, PVM and PVX by ELISA prior to this study. As expected, screening of cDNA libraries only revealed hits for PLRV, PVS and PVY. The sequences obtained for the PLRV-136 isolate were not contaminated with any other virus sequences, confirming that it was a pure isolate of PLRV. The PYV isolate PV-0706 reacted in ELISA only with the PYV (AS-0599) antiserum, and sequencing results revealed hits to three genome components of corresponding ilarviruses (Table 1). These results also confirmed that no additional virus infection was overlooked in this isolate in the previous characterization using only biological and serological methods. All sequencing results are summarized in Table 2.

**Table 1:** Nucleotide and protein sequence identities of PYV to species of the genus *Ilarvirus*.

Virus^a^	Subgroup	RNA 1	RNA 2	RNA 3	MTR/HEL	RdRp	MP	CP
		nt	nt	nt	aa	aa	aa	aa
AgLV	1	43	40	27	35	33	20	11
PMoV	1	43	40	26	34	32	22	11
BCRV	1	42	40	26	34	31	21	11
SNSV	1	42	40	27	34	31	22	10
TSV	1	42	40	26	33	33	20	14
PrRSV	1	42	41	26	33	31	22	13
AV-2	2	43	45	28	35	31	19	14
CVV	2	42	44	26	35	32	15	16
EMoV	2	42	44	29	35	32	19	17
SpLV	2	42	44	29	33	30	19	15
TaMV	2	42	41	28	31	31	18	15
TomNSV	2	42	42	28	33	29	19	14
CiLRS	2	42	42	27	33	31	16	13
ApMV	3	46	44	37	42	34	23	23
PNRSV	3	46	44	36	46	38	25	28
BlShV	3	47	44	37	43	37	27	27
LLCV	3	47	43	35	44	36	25	27
FCILV	4	80	77	72	87	78	86	86
PDV	4	54	55	41	55	50	47	26
APLPV		50	45	33	40	32	20	23
HJLV		46	44	35	42	34	23	16

^a^Ageratum latent virus (AgLV, NC_022128), american plum line pattern virus (APLPV, NC_003452), apple mosaic virus (ApMV, NC_003465), asparagus virus 2 (AV-2, NC_011809), blackberry chlorotic ringspot virus (BCRV, NC_011554), blueberry shock virus (BlShV, NC_022251), citrus leaf rugose virus (CiLRV, NC_003547), citrus variegation virus (CVV, NC_009538), elm mottle virus (EMoV, NC_003568), fragaria chiloensis latent virus (FCILV, NC_006567), humulus japonicus latent virus (HJLV, NC_006065), lilac leaf chlorosis virus (LLCV, NC_025478), parietaria mottle virus (PMoV, NC_005849), privet ringsport virus (PrRSV, NC_027929), prune dwarf virus (PDV, NC_008037), prunus necrotic ringspot virus (PNRSV, NC_004363), spinach latent virus (SpLV, NC_003809), strawberry necrotic shock virus (SNSV, NC_008707), tobacco streak virus (TSV, NC_003842), tomato necrotic streak virus (TomNSV, KT779205), tulare apple mosaic virus (TaMV, NC_003834)

**Table 2:** Comparison of different library preparation methods regarding the number of reads mapped to the respective viral sequences.

Illumina library No.	Virus isolate	Method for library prepartion	Virus source	Total No. of reads	Reference genomes determined this study	No. of reads mapped to reference (MTR) genome	Max Coverage	% reads MTR genome	Virus Size nt	% reads MTR genome/ Kb
Library-01	W13-136	total RNA (ribominus)	Fresh leaf	3,770,990	PLRV	4,002	306	**0.1**	5,883	0.018
					PVY	76,811	3,588	**2.0**	9,703	0.209
					PVS	269,237	21,812	**7.1**	8,485	0.841
					all	350,050	n.d.	**9.2**		
Library-02	W13-136	total RNA (ribominus)	Fresh leaf	1,681,458	PLRV	1,710	131	**0.1**	5,883	0.017
					PVY	11,236	551	**0.7**	9,703	0.069
					PVS	54,323	3,980	**3.2**	8,485	0.381
					all	67,269	n.d.	**4.0**		
Library-03	W13-136	IC-1 (PLRV, PVY, PVS)	Fresh leaf	2,220,836	PLRV	19,380	2,281	**0.9**	5,883	0.148
					PVY	525,810	16,723	**23.7**	9,703	2.440
					PVS	357,645	27,714	**16.1**	8,485	1.898
					all	902,835	n.d.	**40.7**		
Library-04	PLRV-136	Total RNA (ribominus)	Leaf (-20°C)	4,152,506	PLRV	2,052	92	**0.05**	5,883	0.008
Library-05	PLRV-136	IC-1 (PLRV)	Leaf (-20°C)	2,058,564	PLRV	16,714	2,318	**0.8**	5,883	0.138
Library-06	PLRV-136	IC-1 (PLRV)	Fresh leaf	2,002,016	PLRV	26,541	3,384	**1.3**	5,883	0.225
Library-07	PLRV-136	IC-1 (PLRV)	ELISA sap (-20°C)	1,163,612	PLRV	29,179	3,879	**2.5**	5,883	0.426
Library-08	PV-0706	Total RNA (ribominus)	Freez dry	6,169,938	PYV RNA-1	128,647	10,283	**2.1**	3,467	0.601
					PYV RNA-2	101,889	8,105	**1.7**	2,567	0.643
					PYV RNA-3	476,438	48,871	**7.2**	2,375	3.251
					all	706,974	n.d.	**11**		
Library-09	PV-0706	IC-1 (PYV)	Freez dry	1,801,708	PYV RNA-1	4,907	885	**0.3**	3,467	0.079
					PYV RNA-2	4,156	773	**0.2**	2,567	0.090
					PYV RNA-3	6,521	1642	**0.4**	2,375	0.152
					all	15,584	n.d.	**0.9**		
Library-10	PV-0706	IC-2 (PYV)	Freez dry	1,185,478	PYV RNA-1	6	n.d.			
					PYV RNA-2	2	n.d.			
					PYV RNA-3	10	n.d.			
					all	18	n.d.			

### Sequence analysis of PLRV, PVY and PVS

The full-length genome sequence of PLRV from W13-136 (accession number MH937415) was assembled from the final sequence obtained from the de novo assembly (Library-01) and from the sequencing of RT-PCR products from 5’- and 3’-RACE reactions for the same RNA extract. The full-length genome sequence of PLRV (5883 nt) represented a typical *Polerovirus*, showing the closest nucleotide BLAST hit to PLRV isolate PBI-6 (accession number JQ420903, query cover 100%, identity 99%) originating from potato in India.

The PVY from the mixed infected W13-136 sample (accession number MH937417) was assembled de novo from Library-01 reads with a complete genome sequence of 9703 nt. The top nucleotide BLAST hit (100% coverage, identity 99%) was to PVY isolate F65 (accession number KX184818) from potato from Israel.

The complete genome sequence of the PVS isolate (accession number MH937416) was determined to be 8485 nt. PVS W13-136 presented the typical genome organization of carlaviruses. The top GenBank hit found by BLAST analysis was to PVS isolate 89.249 (accession number HF571059, query cover 100%, nucleotide identity 98%) from *S*. *tuberosum* from Hungary.

### Sequence analysis of PYV

**Fig 1:**
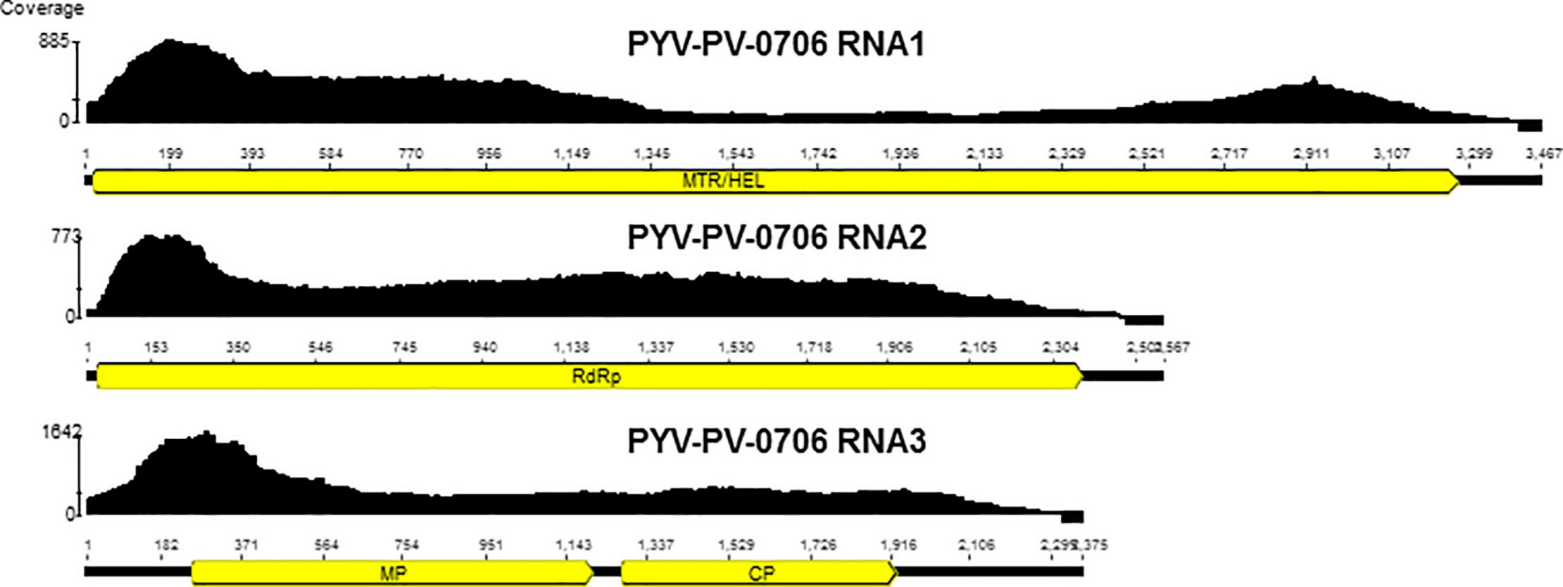
PYV genome organization and coverage with reads from Library-09.

**Fig 2:**
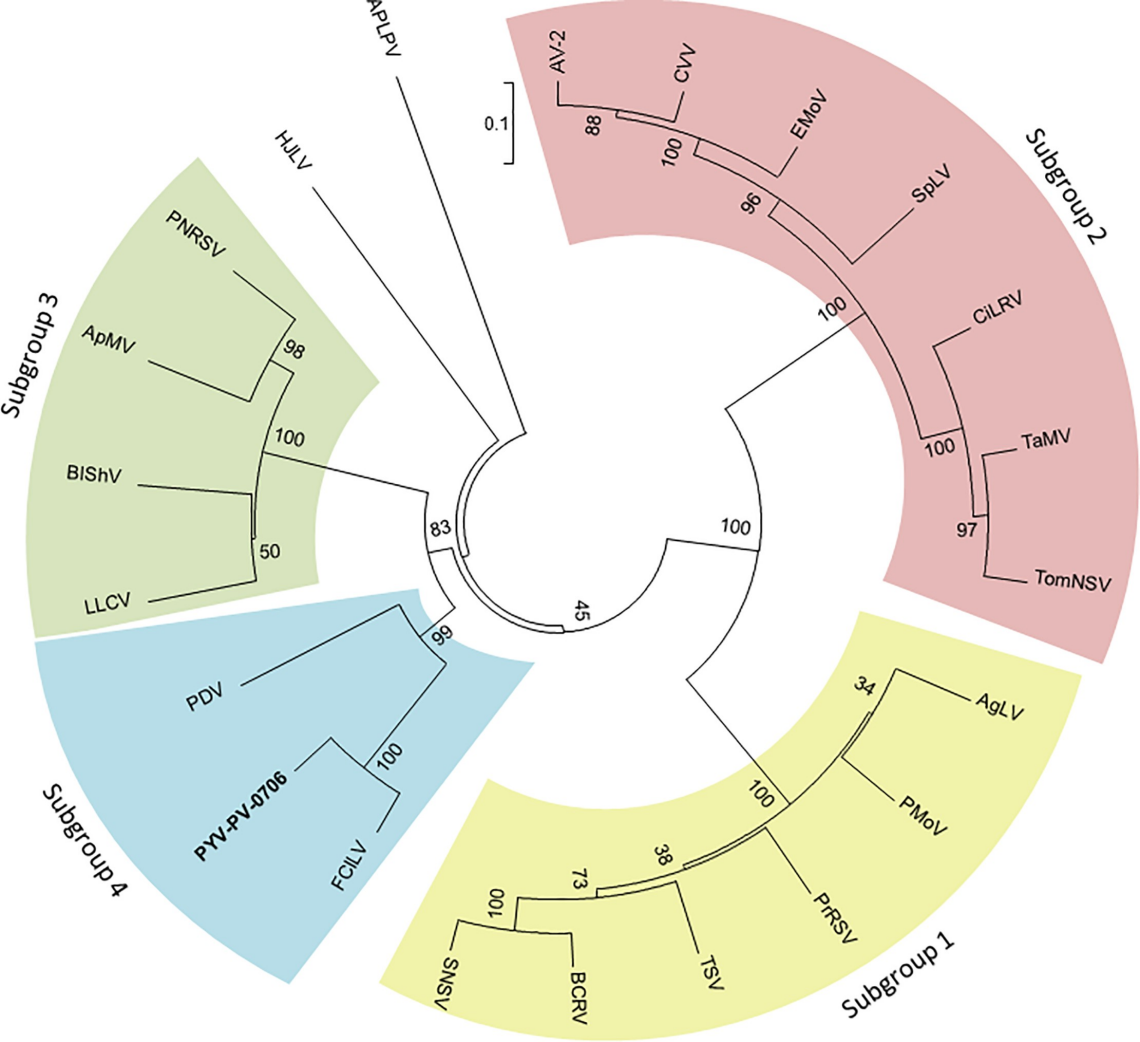
Phylogenetic tree based on RdRp amino acid sequence alignment of members of the genus *Ilarvirus*. Bootstrap values (%) are shown at the nodes. Virus names and GenBank accession numbers are given in Table 1.

Three viral genome components, referred to as RNA1, RNA2 and RNA3, of PYV isolate PV-0706 were assembled from Library-09. The 5’ terminus of each of the genome components was determined by 5’ RACE from cDNAs tailed for each of the genome components with G, C or T in separate reactions. For determination of the 3’ termini, tailing of total RNA was achieved with poly(A) polymerase for all four ribonucleotides to synthesize homopolymers prior to 3’ RACE. Analysis of the 3’ RACE sequences revealed a stretch of identical 100 nt sequences among the three RNAs, with only minor differences in the terminal 170 nt. The lengths of the PYV genome component were determined to be 3467 nts for RNA1 (MH937418), 2567 nts for RNA2 (MH937419) and 2375 nts for RNA3 (MH937420) (Fig 1). RNA1 and RNA2 each encoded a single large ORF. The putative protein encoded by RNA1 showed methyltransferase and helicase motifs, whereas in the RNA2-encoded protein, RNA-dependent RNA polymerase motifs could be identified. For RNA3, two ORFs could be predicted. The 5' terminal ORF showed the highest similarities to movement proteins, whereas the 3' terminal ORF showed similarities to *Ilarvirus* coat proteins. Overall, a genomic organization typical of ilarviruses could be inferred. When compared to the sequences available in GenBank, all three components showed the top nucleotide BLAST hits to *Fragaria chiloensis* latent virus (FCILV) isolate CFRA 9087 (NC_006566, NC_006567 and NC_006568), a member of subgroup 4 in the genus *Ilarvirus* (family *Bromoviridae*). The pairwise identity values ranged from 72% to 80% (Table 1). When the amino acid sequences of the four predicted open reading frames (ORF) were compared to FCILV, the identities ranged from 78% to 87%. As expected, phylogenetic analysis of the PYV RdRp amino acid sequence showed clustering with the other subgroup 4 ilarviruses (Fig 2).

### Comparison of library preparation methods based on total RNA and IC

Two library preparation methods, one starting with rRNA-depleted total RNA, followed by cDNA synthesis, and the other starting with IC, followed by cDNA synthesis, were compared in three different settings. In the first setting, fresh potato leaf material of isolate W13-126, infected with PLRV, PVY and PVS, was used. In the second setting, fresh or frozen potato leaf material of PLRV-136 was analyzed, and in the third setting, freeze-dried *D*. *stramonium* tissue infected with PYV isolate PV-0706 was investigated. The different settings and sequencing results are summarized in Table 2.

In the first setting, when the libraries were generated from fresh leaf material of virus isolate W13-136, two independent repetitions (Library-01 and Library-02) prepared from total RNA were compared with a library prepared by the IC method. For the libraries based on RNA, in both cases, approx. 0.1% of the reads mapped to a PLRV reference sequence. In contrast, the library prepared via the IC method (Library-03) resulted in 0.9% of reads mapping to PLRV (Table 2). A similar trend was observed for PVY, with percentages ranging from 0.7% (Library-02, total RNA) to 2.0% (Library-01, total RNA), whereas the library prepared via the IC method showed a significantly higher percentage of 23.7% virus-specific sequences (Library-03). For PVS, the percentages of reads that mapped to the viral genome were 3.2% and 7.1%, compared to 16.1% for the library prepared by IC. In summary, the total percentages of viral reads in setting one for potato sample W13-136 were 4.0% and 9.2% for the sequencing of total RNA, whereas the IC library showed an unambiguously higher percentage value of 40.7% of total virus-specific reads (Table 2).

In the second setting, the libraries were prepared from plant material harboring virus isolate PLRV-136 stored at -20°C. Library-04, prepared from total RNA, exhibited 0.05% viral reads, whereas enrichment via IC (Library-05) resulted in 0.8% of PLRV-specific reads. When fresh leaf material was used for IC, the viral hits for PLRV reached 1.3% (Library-06), and IC from ELISA sap stored at -20°C resulted in 2.5% (Library-07, Table 2).

In the third setting, libraries were prepared from freeze-dried leaf material harboring PYV isolate PV-0706. Here, the viral hits for all three genome components of PYV reached 11% of reads that mapped to the PYV genome for the library prepared from total RNA (Library-08), whereas the library prepared by IC (Library-09, simultaneous incubation of beads with IgGs and plant extract) reached only 0.9% (Table 2). When using the second IC method (IC-2, sequential incubation first with IgGs, followed by plant extract), the viral hits were close to zero (Library-10). In contrast to the first two settings, for setting three, the overall percentage of viral reads was significantly higher for the library prepared from total RNA compared to that prepared via IC.

## Discussion

The focus of this study was to compare strategies for enriching plant extracts for virus sequences prior to HTS. For this purpose, a potato sample that was mixedly infected with PVY, PVS and PLRV and a plant virus sample (PV-0706) containing an isolate of PYV (a virus for which only a partial genome sequence was available) were analyzed. The genome sequences of all virus isolates were assembled from cDNA libraries prepared from total RNA or from IC templates. The obtained PYV, PVS and PLRV sequences showed only marginal sequence variations compared to already published virus sequences, with nt identities ranging from 98% to 99%.

PYV was first found in potato plants from Peru and was initially described as Andean potato yellowing virus without providing sequence information. The virus was later renamed potato yellowing virus [29, 30, 31]. In subsequent studies, PYV was reported on potato and pepper plants from Chile and Ecuador with only partial sequence information provided [32, 33] covering less than 1 kb of the genome. Our PYV isolate (PV-0706) was obtained from a pepino fruit (*Solanum muricatum*) of unknown geographic origin purchased in a German supermarket and was propagated on *D*. *stramonium*. The partial sequences available (RNA1: KM244740, RNA2: KT160434 and KP996592) were almost 100% identical to our newly determined genome sequences. From these data and the complete genome sequence determined in this study, PYV can be confidently assigned as a distinct species in genus *Ilarvirus* that is most closely related to FCILV. The availability of genome sequences will allow the further development of sequence-based detection and identification methods, which is important because PYV is listed in EPPO Annex A1, comprising pests recommended for regulation as quarantine pests of the EU (https://www.eppo.int/QUARANTINE/listA1.htm) [34].

In this study, we report a new method for enriching viral templates for the preparation of HTS libraries by using immunocapture (IC) to trap virions in a pull-down assay. The maximum enrichment achieved via the IC method using virus-specific antibodies was 40%, compared to a virus purification protocol that achieved 89% enrichment for viral sequences [7]. However, virus purification protocols are costly and time-consuming, and it is questionable whether viruses in mixed infections will be purified simultaneously. For IC, only minute amounts (e.g., 100 mg) are required, similar to those needed for RNA extraction, which enables library preparation from specific leaves or sections thereof. According to Blouin *et al*. 2016, the enrichment efficiency achieved through the IC of dsRNA is between 31–74% of reads of viral origin. However, the possibility of enriching viral dsRNA by using an antibody depends on whether the virus infections result in considerable quantities of dsRNA intermediates. This might not be the case for all viruses and result in an underrepresentation of virus-derived sequences in HTS. Blouin *et al*., 2016, proposed the use of a PCR enrichment step to accumulate sufficient DNA for library preparation [35]. This PCR enrichment can, however, lead to a bias toward viral sequences and the introduction of sequence variation. In contrast, enrichment for viral sequences with virus-specific antibodies depends only on the specificity of the antiserum used, and cDNA is synthesized from unaltered genome sequences. The presented IC method for enrichment assumes that the virus species is known prior to the investigation and that the corresponding specific antibody is available. This is certainly a limiting factor that excludes the use of this method for specific applications.

Other viral enrichment strategies for HTS such as the enrichment of polyadenylated RNA for potyviruses, RCA for circular DNA viruses or the use of small RNA extracts result in percentages of approximately 10%, 60% or 30% of viral reads, respectively [26, 1, 36]. In a recent project the ability of 21 laboratories to detect 12 plant viruses in datasets of small RNA sequences was assessed. This study revealed some essential aspects. Independent from the viral enrichment strategy, the successful identification of viruses depended on factors like the sequencing depth, the bioinformatics strategy and the level of scientific expertise [37].

In our study, HTS libraries from IC preparations and from rRNA-depleted total RNA were compared for four virus species (Table 2). Based on the literature, for total RNA extracts lacking ribosomal RNA depletion, the percentage of viral reads for HTS is reported to range from 0.5% to 2% [17, 38, 39]. By removing ribosomal RNA prior to library preparation, between 0.02% and 6% virus-specific reads can be obtained [9, 11, 40, 41]. In our study, between 0.05% and 11% virus-specific reads could be obtained (Table 1). However, the lowest values were observed for PLRV, ranging from only 0.05% to 0.1%. This result may be explained by the restriction of poleroviruses to phloem tissue [42, 43], limiting the number of viral reads. With the IC method, the percentage of viral sequences obtained for PLRV could therefore be increased tenfold. A similar trend was observed for PVY and PVS. In general, the enrichment for viral reads ranged from 2 to more than 30 times higher for IC compared with rRNA-depleted total RNA. However, for PYV sequencing, the opposite situation was observed. Here, the percentage of viral reads under the RNA-based method (Library-08) was more than 10 times higher than that under the IC method using virus-specific antibodies. A possible explanation for this disparity could be the low binding affinity of the antibody or low virion stability (freeze-dried leaf material was used), limiting the binding of intact RNA containing virions to the capture antibody. In contrast, virions of poleroviruses are known to be insensitive to freezing [42], which could provide an explanation for the contrary results obtained for PLRV. We obtained good enrichment using the IC method for PLRV, a virus that due to its phloem limitation, represents a typical low-titer virus. Good virion stability provides a basis for the efficient binding of intact virions with viral RNA, serving as a template for HTS. In general, high virus coverage is required for viral population studies, where the coverage should exceed 10,000 [7]. This coverage is achieved in many studies by the amplicon sequencing of partial genome sequences. However, an amplification step can result in additional bias [7]. This situation can ideally be avoided by using the presented IC method, as shown by the PVY and PVS examples. Additionally, the entire genome can be analyzed.

With the IC method, we provide an advantageous way to efficiently enrich viral sequences prior to library preparation for HTS. This method is an option for enriching specifically for viruses, and when several antibodies are combined, a broader spectrum of species can be covered. However, the enrichment efficiency depends on the antibodies used and may be affected by virion stability.
